# Altitude Training and its Influence on Physical Endurance in Swimmers

**DOI:** 10.2478/v10078-011-0026-9

**Published:** 2011-07-04

**Authors:** Marek Strzała, Andrzej Ostrowski, Zbigniew Szyguła

**Affiliations:** 1 Department of Theory and Methodology of Water Sports, University School of Physical Education, Cracow, Poland; 2 Department of Theory and Methodology of Water Sports, University School of Physical Education, Cracow, Poland; 3 Department of Sports Medicine, University School of Physical Education, Cracow, Poland

**Keywords:** altitude, hypoxia, training, swimming

## Abstract

It is possible to plan an altitude training (AT) period in such a way that the enhanced physical endurance obtained as a result of adaptation to hypoxia will appear and can be used to improve performance in competition. Yet finding rationales for usage of AT in highly trained swimmers is problematic. In practice AT, in its various forms, is still controversial, and an objective review of research concentrating on the advantages and disadvantages of AT has been presented in several scientific publications, including in no small part the observations of swimmers. The aim of this article is to review the various methods and present both the advantageous and unfavourable physiological changes that occur in athletes as a result of AT. Moreover, AT results in the sport of swimming have been collected. They include an approach towards primary models of altitude/hypoxic training: live high + train high, live high + train low, live low + train high, as well as subsequent methods: Intermittent Hypoxic Exposure (IHE) and Intermittent Hypoxic Training (IHT). Apnoea training, which is descended from freediving, is also mentioned, and which can be used with, or as a substitute for, the well-known IHE or IHT methods. In conclusion, swimmers who train using hypoxia may be among the best-trained athletes, and that even a slight improvement in physical endurance might result in the shortening of a swimming time in a given competition, and the achievement of a personal best, which is hard to obtain by normal training methods, when the personal results of the swimmer have reached a plateau.

## Introduction

Altitude training (AT) is used by athletes in order to improve their physical endurance and to increase their endurance and movement economics after they have returned to sea level. This practice has been used for decades. Different environmental conditions with a reduction in ambient oxygen cause adaptive changes to sportsmen’s organisms, which can be described as “natural doping”. The process of metabolic adaptation to a changed environment takes place in tissues at the cellular level without the necessity of drug intake. Physical exercise performed in hypoxia causes amplified synthesis of the HIF-1 factor (hypoxia inducible factor 1) influencing messenger RNA coding that determines the excretion of enzymes that are involved in muscle energy producing processes. In this way, muscle angiogenesis is also controlled by increasing the level of vascular endothelial growth factor (VEGF) (Lundby et al., 2009). HIF-1 also regulates the expression of the gene responsible for erythropoietin (EPO); it also regulates the organism’s pH and glycolysis (Vogt et al. 2010). The cognizance of advantageous physiological changes in an organism following hypoxia ensures that athletes readily use AT. In sport, there is a belief that it is possible to plan an AT period in such a way that the enhanced endurance obtained as a result of adaptation to hypoxia will appear after the athlete has returned from the mountains and can benefit competition. Many observations conducted during hypoxic training, which were initiated in the 1960s during preparation for the Olympic competition in Mexico City in 1968, which is located 2300 m above sea level, encouraged coaches and athletes to apply AT.

Yet finding unequivocal rationales for undertaking altitude training, is problematic. In practice, AT and its various forms are still controversial, and an objective review of research concentrating on AT advantages and disadvantages has been presented in several scientific publications, including several observations in swimmers. The main problem of those AT observations that have been conducted is the lack of results that were replicated in similar experiments. In the natural conditions of high mountains, there is a multitude of changing conditions, such as humidity, insolation, atmospheric pressure, that affect adaptation. In addition, in different procedures using surrounding hypoxia, other poorly controllable factors play an important role in the athletes adaptive changes. These include: the diet used, which influences water-electrolyte equilibrium and iron intake, training intensity and appropriate recovery duration. One drawback of the large number of observations noting advantageous adaptive changes is that there has been little variety when it comes to parallel comparative research conducted on a homogenous group of athletes in normoxia and hypoxia.

The aim of this article is to review the various methods and present the advantageous and unfavourable physiological changes occurring in the organisms of athletes as a result of AT. Moreover, AT results in the sport of swimming have been collected and presented.

### Gene expression caused by hypoxia

In recent years, scientists have been attempting to answer the question, in what way does hypoxia influence gene expression? In Semenza’s research (2000), it has been noted that the main factor regulating aerobic homeostasis and plays an important role in the cardio-vascular and ventilatory response to hypoxia is the earlier mentioned hypoxia inducible factor 1 (HIF-1), which is present in all the nucleated cells of our organism. The presence of this factor is regulated by oxygen concentration in the cells. In kidney cells, synthesizing EPO influences the erythropoietin gene transcription and increases its excretion, causing a rise in red blood cell production during hypoxic exposure. HIF-1, which is present in many cellular lines of the organism, streamlines the action of glycolytic enzymes (phosphofructokinase, hexokinase and lactate dehydrogenase) taking part in exercise metabolism. It contributes to the regulation of transport and the utilization of glucose with the participation of glucose-1–4 transporters and lactate metabolism in muscles, with the involvement of carbonic anhydrase (Lundby, 2009). Moreover, this factor in hypoxic training influences the process of increasing the buffering base capacity (Gore et al., 2001) and induces more efficient oxygen utilization in working muscles (Gore et al., 2007).

When considering the possibility of physical endurance being enhanced as a result of AT and associated hypoxia, it should be noted that AT promotes an increase in anaerobic glycolytic metabolism. Such statistically significant anaerobic work indices were noted in research on competitive swimmers (Martino et al., 1995; Nomura et al., 1999; and others). During intensive physical exercises, the struggle is accompanied by the production of large amounts of lactate, and the maintenance of an effective exercise metabolism depends on the efficient transport of the produced lactate and H^+^ ions. Transport within skeletal muscle is controlled through cell walls from muscle to blood using two transporters – monocarboxylate MCT1 and MCT4. That is why an increase in the level of the proteins in muscular tissue as a result of endurance AT can minimize exercise pH fluctuations (Gore et al., 2007). In the research of Zoll et al. (2006) a 44% increase in MCT1 concentration in the muscle of 9 well-trained runners after a six-week training period in hypoxia in a simulated altitude of 3000m was noted in comparison to a control group. In this research, the post-experiment time of a run to exhaustion at the intensity responding to 100% V̇O_2max_ was registered, as well as a simultaneous increase of post-exercise blood LA concentration when compared to the same effort before hypoxia. It was stated that the MCT1 concentration increase enabled the improvement of anaerobic metabolism and the removal of lactate from cells, which caused a slower decrease of pH at the same race pace. A correlation between MCT1 expression in mRNA and running results after hypoxic training at the level of r=0.60 was also noted. The possibility of adjusting the concentration of H^+^ ions in muscular tissue strongly depends on buffering bases. Their increased concentration might belong to one of the mechanisms responsible for enhanced performance as a result of AT (Gore et al., 2001; Mizuno et al., 2008).

### Haematological and cardiovascular-respiratory adaptation

AT and its variants are used in sports training mainly for the purpose of gaining better haematological indices that influence endurance capabilities during prolonged effort. Because lower oxygen partial pressure (P_I_O_2_) in breathing air causes increased EPO production, kidney artery blood oxygenation decreases. Endogenously produced EPO influences bone marrow stem cells, increasing red blood cell-line cells precursors, which consequently cause an increase in erythrocyte production (Jelkmann, 1992). Research conducted on well-trained swimmers, runners and triathlonists in different hypoxic conditions did not always show this kind of relationship (Nomura et al., 1999; Friedmann et al., 2005; Robach et al., 2006; Gore et al., 2006). Some research indicates that subjects who reside in mountainous areas and descend to sea level, have intensified HIF-1 decomposition (Jelkmann, 2004) and consequently there is a decrease in EPO synthesis. This can influence the reduction of erythrocytes precursors and decrease the vitality of red blood cells. This phenomenon is called neocytolysis (Rice et al., 2005; Risso et al., 2007). In other research it has been observed that after 1–2 days at altitude, blood EPO level reaches its peak value and then it progressively decreases to base level (Eckardt et al., 1990). In their observations Robach et al. (2004) noted an increase in sTfR level and a simultaneous decrease in the EPO level during a one-week stay at an altitude of 4300 m above sea level, then EPO concentration decreased to basic values in 24h after the return to sea level.

*Inter alia* that is why in the examination of athletes, in which erythropoiesis effects, such as an increase in the erythrocytes mass (RCV) or the percentage of blood haemoglobin, iron status was additionally controlled with the simultaneous determination of blood plasma transferine receptors (sTfR). Blood plasma sTfR concentration correlates strongly with the number of cell erythroblast receptors – TfR. In the human organism, iron delivery to numerous in bone marrow erythroblasts is enabled by the interaction between transferine in blood plasma and surface TfR receptors (glycoprotein membrane regulating cellular iron intake from blood circulation) positioned on erythroblasts (Mierzwa et al., 2006). Proliferating and maturing erythroblasts accumulate haemoglobin and synthesise haem molecules using iron; these iron resources simultaneously start to be reduced, and if the organism lacks iron it increases the sTfR level (Beguin, 1992). That is why in research that has monitored the hematopoetic mechanisms in athletes, (e.g. Koistinen et al. (2000) and Robach et al. (2004)) a mutual dependence between sTfR receptors level and erythropoiesis was noted. An effect of the processes described above is an increasing number of red blood cells and erythrocytic mass (polycytaemia), and further growth in the amount of haemoglobin, which leads to the improvement of aerobic metabolism (Schmitt et al., 2006).

Hypoxic-hypobaric surrounding conditions promote respiratory adaptation, which manifests itself in the improvement of maximal minute ventilation (V̇_E_max). Such a statistically significant increase in V̇_E_ max was noted in research on swimmers (Truijens et al., 2003; Rodríguez et al., 2007). These changes, together with the improvement of oxygen transport, enhance cardiovascular-respiratory endurance, assessed by V̇O_2max_. Greater oxygen utilization enables a more efficient aerobic muscular energy production in prolonged physical efforts.

### Exercise metabolism enhancement

For well trained athletes, a favourable phenomenon is the enhancement of movement economics combined with a reduction in oxygen uptake while maintaining the same effort intensity after altitude acclimatization (Green et al., 2000b; Saunders et al., 2009). Oxygen uptake reduction at a given intensity of exercise was associated in those observations with the change of substrate use towards accelerated uptake of carbohydrates in comparison to free fatty acids, which was supported by the level of respiratory indices: an increase in the RER index without changing V̇CO_2_ and lowering V̇O_2_. In this situation, an increase in energy production – ATP per mole of oxygen used in full oxidation of glycogen (Green et al., 2000b) – was obtained.

### Unfavourable symptoms of AT in athletes

Altitude training in athletes is connected not only with the enhancement of effort efficiency or enhanced physical endurance after a return to sea level. Hypoxic conditions cause additional physiological strain on the organism. Therefore, in order to avoid the build-up of fatigue or acute mountain sickness (AMS), effort intensity is lower in comparison to training at sea level. Wilber (2007a) claims that many runners and swimmers have noticed a loss of race pace fitness form and turnover after specific AT with reduced intensity. Hypoxia lowers muscle energy production with the involvement of free fatty acids in mitochondria in the Krebs cycle, especially at higher effort intensity. The restitution time after training is prolonged; sleeping problems, headaches, nutritional disorders associated with significant atmospheric pressure reduction occur in the first days of altitude acclimatization. More or less intense negative hypoxia effects appear even during the exposition and training at medium heights (Gore et al., 2007); furthermore, swimmers (Miyashita et al., 1998) may suffer from a worse physical condition, including problems such as diarrhea, the common cold, a sore throat or low back pain. It has been noted that at an altitude greater than 2000 m above sea level, AMS symptoms, such as lung and brain swelling, heartbeat distortion and organism immune function weakness, may appear (Bailey et al. 1997). Our knowledge of the trained human organism’s physiological reaction to hypoxic surrounding conditions tends to suggest that what is needed is a reduction of physical exercise intensity, increased recovery after all training units, in order to allow regeneration and progressive acclimatization. It is well known that attempts have been made to maintain the effort intensity close to that of sea level from the very beginning of altitude camps. The will to maintain high training quality without using the base level of training is the main reason as to why Koistinen et al. (1995) have noted an altitude reduction of effort capabilities which is connected to maximal minute oxygen uptake reduction, what especially concerns the best trained athletes, who are characterized by V̇O_2max_ above 65 ml·min^−1^·kg^−1^. Such conditions result in higher vascular hipoxemia and proportionally more reduced work capacity. It is bound to venoarterial shunting, irregular perfusion ventilation and limited alveolar-capillary diffusion.

In the initial phase of staying in the mountains, blood pH changes proceed towards more alkalization together with increased hyperventilation and increased CO_2_ excretion. It induces a lower level of hydrogen ions concentration in body fluids. Active hydrogen ions transportation form blood to cerebrospinal fluid directly without neuron-contacting central control and respiratory system, which lowers blood alkalinity as a result of removing part of the bases by the kidneys (Kozłowski, 1986). Cases of prolonged maintenance of high muscle and blood lactate concentration are caused by an intensified anaerobic metabolism engagement. High lactate and hydrogen ion concentrations might slow the predicted synthesis and excretion of EPO by the kidneys (Gunga et al., 2007) and cause red blood cells haemolysis (Szyguła, 1990), delaying an improvement in haematological parameters. Gore et al. (1998) have noted an overall lowering of creatine concentration in red blood cells in 8 track cyclists after a 31-day stay in the mountains (Toluca, Mexico 2690 m above sea level), and training at different altitudes from 1850 to 4578 m above sea level. The occurrence of these symptoms was explained by an intensification of red blood cell decomposition. In the same cyclists, an initial increase in mean corpuscular volume (MCV) without a corresponding change in Hb mass and blood volume were also found. In research conducted by Wilber et al. (2000), at a high altitude (1860 m above sea level) triathlonists’ conditioning camp, an increase in blood plasma creatine kinase (CK) and cortizol level were noted during and after a 5-week period of intensive training. This is connected with skeletal muscle catabolism (Hakkinen et al., 1989). An increased CK level accompanies muscular tissue microinjuries and the beginning of a delayed onset of muscle soreness. Skeletal muscle microinjuries cause an increase in free intercellular calcium (Ca^2+^) and A_2_ phospholipase. They occur as a result of sarcomer breaks during eccentric contractions (Nosaka et al., 1995). It is also accompanied by post exercise immunosuppression (neutrophillia, eosinophilia, lymphocytopenia) (Nieman et al., 1997) and increased susceptibility to infections.

### Altitude training forms

***LH+TH – live high and train high*** – the original AT method was to continually stay at a medium height between 1500 and 4000 m above sea level; it was assumed that during this period, moderately intense training would result in an increase in blood erythrocyte mass, which means V̇O_2max_ improvement in normoxia conditions, while simultaneously improving endurance (Wilber, 2007a). Live high and train high – LH+TH – for athletes living at sea level takes place during camps in defined periods throughout an annual training cycle. Studies show (Nies et al., 2003) that a good compromise between mountain condition stress and moderate training intensity is the choice of a height of around 2500 m above sea level. Other researchers, considering mainly the haematological indices, which have the strongest influence on endurance capabilities in sports such as running, speed skating, cycling and swimming, the recommended choice of altitude is between 1500 and 3000m (Friedmann et al., 1997). The duration of time spent at AT camps averages 3 weeks (Miyashita et al., 1988; Bailey, 1997; Heinicke at al., 2005), and endurance improvement at sea level might be noted after reacclimatization to sea level conditions, with blood plasma volume alignment, which decreases its volume in high-mountain conditions as a result of increased water excretion (Bailey, 1997).

***LH+TL – live high and train low (via natural/terrestrial altitude)*** – A technique that is well-known and often used by athletes (Wehrlin et al., 2006), the hypoxic training method involves living in natural mountain conditions (high) and training at low altitudes (at sea level). This solution, when compared to the classical *LH+TH* method, enables the maintenance of normal training intensity. It prevents the loss of training power during the initial altitude acclimatization period and enables the activation of favourable altitude effects (cardiovascular, respiratory and metabolic adaptations) (Millet et al., 2010).

***LH+TL – live high and train low by IHE – Intermittent Hypoxic Exposure*** – another solution for hypoxia application is its simulation in normoxia conditions, such as in rooms, tents or chambers (hypobaric hypoxia). This method was introduced by Finnish scientists for their athletes, who wanted to utilise the favourable hypoxia organism adaptation without inconvenient and expensive travelling (Rusko et al., 1995). Athletes are exposed to hypoxia in rooms; training is the only break from the hypoxia. In a hypoxic room, they breath with air depleted in oxygen by N_2_ enrichment (Koistinen et al., 2000; Gore et al., 2001) or some oxygen is filtered out (Robach et al., 2006; Schmitt et al., 2006). These researchers recommend staying at a simulated height of ≥ 3000 m for at least 3h·d^−1^ for 1–3 weeks. Those conditions, in which athletes who train using the IHE method, e.g. swimmers (Rodríguez et al., 2007), closer to a high-mountain climate are those used in hypobaric chambers where a lower atmospheric pressure is present. Rodríguez et al. (2000) suggest that IHE application prevents sport shape decrease after a sudden elevation at significant altitude, and support erythropoiesis with a simultaneous improvement of effort capabilities.

***LL+TH – live low and train high by IHT – Intermittent Hypoxic Training*** – Classified as – *LL+TH (live low and train high)* – living at sea level with altitude training (Wilber, 2007a). This AT model, in which athletes exercise in hypoxic conditions from seconds to hours for periods lasting from days to weeks (Millet et al., 2010). Hypoxia is produced artificially in rooms or hypobaric chambers as well as using hypoxicators, which enable the breathing of a gas mixture (Katayama et al., 2004). This solution was also used in swimmers (Truijens et al., 2003). Such methods simulate the atmospheric conditions present at an altitude of 2500 – 3500 m above sea level. The interval effort in such conditions occurs in periods from 5 to 180 minutes (Wilber, 2007a). Millet et al. (2010) show that intermittent hypoxic interval training interspersed (IHIT) is defined as a method where, during a single training session, there is an alternation between hypoxia and normoxia.

The researchers claim that, in a manner similar to IHE, time spent outside the chamber, in which the IHT method is applied, might also be used for additional normal training activity, as in the case of swimmers in Truijens et al. (2003) and other athletes (Meeuwsen et al., 2001; Hendriksen et al., 2003). Another advantage of the IHT method is recovery after altitude training in sea level conditions, which prevents the occurrence of the negative symptoms of prolonged high-mountain exposure. These circumstances do not force a reduction in the amount of physical training, and they prevent sleep perturbations and dehydration; they also enable normal alimentation. The behaviour of athletes using IHT methods results in the improvement of nonhaematological physical endurance indices, such as an increase in mitochondria density, the muscular fiber of capillary ratio and the cross-section of muscular fibers (Vogt et al., 2001; Czuba et al., 2011). It also enables changes in the blood oxygen transport properties. These effects, however, are not always significant (Truijens et al., 2003; Roels et al., 2007).

***Apnoea*** **–** French researchers (Lemaître et al. 2010) claim that apnoea training may well be a training method used in the future. In sea-mammals, as well as in humans, spleen contraction occurs during apnoea, provoking extrusion of accumulated erythrocytes. In literature studies on this particular field, Lemaître et al. (2010) have observed that in subjects after 5 repeated apnoeas, there is an increase in Hct and Hb (both in the range from 2% to 5%), which reduces arterial oxygen desaturation and enhances exercise metabolism. Lemaître et al. (2010) believe that such effects obtained mainly by freedivers can also be achieved in regular training by swimmers, and it might be an easy method to boost immediate performance. Apnoea should be used directly before a race because its effects (i.e. increased Hct) disappear in 10 minutes after the last apnoea (Espersen et al., 2002; Schagatay et al., 2005). After apnoea training in swimmers, the research conducted by Lemaître et al. (2009) indicated that 30-second apnoea epochs separated by 30 seconds of breathing room air during 1 hour of steady state cycling exercise at 30% of their maximum oxygen uptake presented a significant increase in peak V̇O_2_, minimal arterial oxygen saturation (SaO_2_min) and power at the Respiratory Compensation Point in a maximum incremental test on a cycle ergometer. Swimmers did not improve their performance (clean velocity and time on 50 m); however, they improved their technique by decreasing their stroke rate and increasing their stroke length and index coordination of arm, demonstrating greater propulsive continuity between the two arms action.

### Swimmers altitude training

AT with the use of a chosen hypoxic method is particularly appropriate for the sport of swimming. Former use of the classical method *LH+TH* therefore entailed travelling to a location with a swimming pool situated at the desired height. In swimming AT is designed to enhance the overall physical endurance level in the preparatory phase and obtaining the highest possible sport efficiency shaped in the details for a given competition in the tapering phase. The planned effect to achieve the highest standard of fitness through altitude adaptation is most frequently obtained from 5 to 12 days after returning to sea level at the start of the most important competition phase (Miyashita et al., 1998; Robertson et al., 2010). For example Friedmann et al. (2005) report that the optimum period is 10 days after a return to sea level. According to experienced German coaches this time interval is required for swimmers to achieve maximum sea level performance after altitude training. In this time the results may be influenced by even small changes in haematological and muscle biochemical variables.

Observations conducted on swimmers using various AT methods, beginning with camps using the natural *LH+TH* method, through to artificial approaches using hypoxic chambers or rooms (*LH+TL* or *LL+TH*), or a combined approach that is also used more frequently with swimmers (Table 1a. and 1b.) are described in the literature. From the point of view of subjects who want to apply AT information, how it has been implemented and the effects obtained by other researchers and practitioners of swimming are important.

***LH+TH*** – for example, during two independent three-week camps held in Mexico City (2300 m above sea level) two groups of Japanese swimmers undertook swim training (about 5000 m per two hours) twice a day for successive days and then had a non-training resting day (Miyashita et al., 1988). Researchers qualify that the extent of the swimming distance was in the usual way tapered towards the swim meets three days after altitude training (Table 1a.). The Swim Meet held in Indianapolis 1982 or the Pre-Olympic Games in Los Angeles 1993. The 8 swimmers in 1982 improved their results to a degree that was statistically significant at the distance of 200 m, but no improvement was noticed for the distance of 100 m. In 1993, only 5 of 12-athletes reached very high results at different distances.

In Pyne (1998) observations made at two separate camps in Flagstaff, Arizona (USA) were organized at an altitude of 2100 m above sea level: the first in May 1995 (prior to the Pan Pacific Championships in Atlanta, USA) and the second (prior to the 1996 Olympic Trials in Sydney, Australia) (Table 1a.). The program for the camps consisted of three phases: days 1–5 involved acclimatization, days 6–20 involved volume/intensity training, and days 21–24 involved recovery. Each phase included multiple three day training microcycles. Each day contained two to three swimming sessions. The volume and intensity of training was individualized for each swimmer: the total volume over the camps ranged from 160 to 240 km. In Camp 1, the velocity for the 5^th^ 200 m swim (maximum effort for the 5×200m test) increased (p<0.01) from 1.44±00.4 to 1.48±00.3 m·s^−1^; from day 8 to day 18 at altitude no AT effects on sea level were measured. In Camp 2 a significant improvement in swimming velocity was noted by 00.2 m·s^−1^ from day 8 to day 18 and from pre 1.49 to 1.53 m·s^−1^ post-altitude.

Roels et al. (2006) subjected the swimmers to a *LH+TH* procedure during the two camps at different altitudes (Table 1b.). In those periods, swim training at altitudes of 1200 m and 1850 m above sea level included 86% and 84% effort with an intensity equal or below the OBLA threshold respectively. During the stay at a greater height maximum minute heart rate decreased in a way that was statistically significant, from 188.0±10.4 to 181.0±6.8 bts·min^−1^ and blood LA in a graded test until volitional refusal, in comparison to the level before hypoxia, from 7.1±1.6 to 6.4±1.9 mmol·l^−1^. Whereas after the stay at an altitude of 1850m, a larger increase and higher level of sTfR in athletes’ blood was noted. The swimming speed in the endurance test at a distance of 2000m front crawl improved only after training at an altitude of 1200m, in parallel to a statistically significant increase in the arm stroke rate (SR) from 32.6±3.6 to 33.9±3.2 cycle.min^−1^ and a significant decrease in arm stroke length (SL) from 2.29±0.25 to 2.15±0.19 m. The effects of swimming speed improvement at the distance of 2000m (from 1471±35 to 1442±45s; p<0.05) after the *LH+TH* camp at an altitude of 1200m in a slightly modified group of swimmers, but in the same circumstances, were described by the same research team in another paper (Schmitt et al., 2006).

***IHT*** – in artificially developed hypoxia conditions the organism of the swimmer is passively exposed, or by using appropriate equipment, such as flume training. In a specially designed chamber in Ogita et al. (1999) research on IHT training included, among other things, 7–8 repetitions x 20 seconds of swimming with an intensity of 130% V̇O_2max_ with 10-second rest periods (Table 1a.). Ogita (2006) points out that high intensity intermittent or endurance training in hypoxia is a good way to enhance anaerobic properties measured by maximum accumulated O_2_ deficit (MAOD) and aerobic endurance level (V̇O_2max_). Such training in hypoxia mostly develops the anaerobic component, which plays an important role in 100–200 m distance swimming (Ogita, 2006). Observations were conducted by his team on two groups of swimmers: the Control group (C) trained under normal conditions and the Hypoxic group (H) exercised under hypoxic conditions that simulated an atmospheric pressure of 1600 and 2400 m above sea level (Table 1.a). Both groups conducted three types of high intensity intermittent or endurance training; 1) a 2-min bout at OBLA separated by 15-s recovery were repeated 15 times, 2) a 2 min bout at 50% V̇O_2max_ and a 3 min bout at 100% V̇O_2max_ were continuously repeated 5 times, 3) a 20-s bout at 170% V̇O_2max_ separated by 10s recovery was conducted for at least eight sets or more. Training sessions 1) and 2) were undertaken in the hypobaric condition that corresponded to 1600 m above sea level, and training 3) was conducted at 2400 m above sea level. Before and after the training period, V̇O_2max_ and MAOD (maximum accumulated O_2_ deficit), as well as swimming performance at 100m and 200m free style were determined. After 3 weeks of training, mean values of V̇O_2max_ increased significantly from 56 to 62 ml·kg^−1^·min^−1^ in group C, and from 56 to 63 ml·kg^−1^·min^−1^ in H, what amounts to 12%. MAOD increased significantly from 61 to 70 ml kg^−1^ in C, and from 56 to 72 ml ·kg^−1^ in H respectively. When the increased ratio of MAOD between the two groups were compared, it was significantly greater in group H (29%). The swimming performance at the distances of 100 m and 200 m was significantly improved in both groups: in 100 m from 55.86±1.44 to 55.09±1.71 seconds in group H and from 56.92±1.81 to 56.09±1.71 seconds in group C. At the 200 m distance, the improvement equaled from 121,27±2.27 to 119.27±2.37 and from 123.68±2.62 to 121.26±3.03 in groups H and C respectively (Ogita, 2006).

The observations of Truijens et al. (2003) (Table 1a.) were conducted on a swimmers experimental group (IHT) and a control, which unconsciously breathed normal air, during the same interval training: 10 × 30 seconds of effort with a 15-second recovery, then 5 × 1-minute effort with a 30-second recovery and then 5 × 30-second effort with a 15-second recovery. Between the series a 2-min recovery was used. All subjects completed a minimum of 12 flume training sessions; the hypoxic group trained with significantly reduced V̇O_2_ in comparison to the normoxic group (hypoxic group: 69.0±13.9% of pre-training V̇O_2max_ for 30 s, and 75.7±9.2% for 1-min sessions; normoxic group: 90.9±10.3% for 30 s, and 93.8±11.5% for 1-min sessions). Furthermore, the power output during training, expressed as a percentage of maximum power output as determined on the pretest, was significantly lower, by 7.2±7.3% in hypoxia (Truijens et al., 2003). During the individual series, the water flow speed in the channel was increased from 0.03 to 0.05 [m·s^−1^] in such a way that the athletes were strongly encouraged to finish the effort that approached the extent of their capabilities. Depending on the group, swimmers breathed through a specially designed mouthpiece either normal air (the placebo group) or oxygen-depleted air to FIO_2_ 15.3±0.1% (the hypoxic group). All out-of-flume training sessions were rated as either of low or moderate intensity: between 35 and 46% of the out-of-flume sessions per week were rated as low-intensity training sessions with the remainder rated as moderate. During this research the swimming speed and V̇O_2max_ after control training and IHT training improved in both groups.

***IHE*** **–** in observations conducted by Robach et al. (2006) (Table 1b.) on the *LH+TL* procedure covered the living and training of experimental and a control groups at an altitude of 1200 m above sea level in Prémanon (France). The training profile of the two groups of swimmers included: 85% of training at or below OBLA (intensity I and II with LA 2–4 mmol·l^−1^), 13% of training at or above OBLA (mostly intensity III LA between 4–6 mmol·l^−1^ and IV LA 6–10 mmol·l^−1^) and 2% with speed and weight-lifting exercises (LA up to 16 mmol·l^−1^). Despite a significant increase of total haemoglobin mass (THM) of ∼7% on average and red blood cell volume (VRBC) 8,5% no correlation was found between the changes V_RBC_ in and the concurrent changes in V̇O_2max_,which increased close to significance in case of V̇O_2max_ (6.9%). These researchers claim, however, that it cannot totally exclude the possibility of a leak during the rebreathing period at some point in these subjects, leading to a loss of CO and, therefore, an overestimation of their THM. No improvement was observed in 4×200 or 2000 m all out tests. In swimmers in the control group, training at an altitude of 1200 m improved their performance in the 2000 m test as well as in the incremental test speed after 15 days.

In the Rodríguez et al. (2007) examination of the 3h per day hypoxic-hypobaric exposure, from Monday to Friday, in the competitive period (Table 1b) enabled the implementation of a normal swimmers’ day schedule during 4 weeks and their participation in competitions. Both groups, an experimental and a randomly chosen “blind” group, spent most of their time in conditions corresponding to an altitude of 500 m above sea level; they did, in terms of volume, similar training of 10.2±2.0 hours a week with 4±3 intensive training units. Only in the experimental group in the third week of IHE was a significant increase in physical endurance indices observed (Table 1b.); and the front crawl swimming speed at a distance of 400m, although it improved by 2.3 seconds, the difference remained statistically insignificant. This minor effect may encourage the implementation of IHE because it was achieved together with V̇O_2max_ increase by 0.3 l.min^−1^ 3 weeks after hypoxic intervention and following precompetition tapering. These experimental results concerning other endurance indices and economics of swimming with submaximum speed of swimmers were presented in other works by the same authors (Truijens et al., 2008). No significant increase in results was noted. It is remarkable that Rodríguez et al. (2003) noted more favourable effects of a 2-week IHE in swimmers (Table 1b.).

**Combined** – in Friedmann et al. (2005) research the training included 60–70 km of swimming per week, mainly endurance, below the OBLA threshold (55%), with 9% participation of training intensity corresponding to lactate changes in the organism at the level of 4–6 mmol.l^−1^. 30% of the training was concentrated on perfecting a given swimming style. The erythropoietin increase (10– 185%) after 4 hours of exposure to normobaric hypoxia showed considerable inter-individual variation and there was a significant correlation (p<0.001) with the acute erythropoietin increase during altitude training but not with the change in THM (a significant increase of ∼6% on average). The change in sea level performance after altitude training was not related to the change in THM (Friedmann et al., 2005).

In Robertson et al. (2010) (Table 1b.), swimmers in a 5-month period had three to four 10-day hypoxic stimulations (*LH+TH*): in the 1^st^ and 3^rd^ phase swimmers stayed for 9–10 hours at night in normobaric tents or chambers (IHE) and the rest of the day trained in Canberra (Australia) at an altitude of 600m. Between the 10-day blocks there was a 4–5-week gap. In the 2^nd^ and 4^th^ phases the athletes underwent training in the same conditions as in first 5 days of the 1^st^ and 3^rd^ phases; they then used *LH+TH* at an altitude of 1350 m above sea level in Thredbo (Australia). On average, in each of the 4 hypoxic blocks athletes gained small, but according to the authors, significant, improvement of total haemoglobin mass, by 0.9% on average, and swimming speed at OBLA intensity also rose by 0.9%. A small improvement was obtained in the endurance test results for freestyle 2000 m and the 1200 m breaststroke swimming race by 2.4±2.0%, which substantially increased after block 2: 2.3±1.7% at r=0.47 was noted. Measurements of the most important variables determining swimming efficiency in the hypoxic group showed a small increase; they can, however, be particularly significant for swimming competitions. However, the lack of enhanced essential swimming results in the most important national competition as well as in the Commonwealth Games after blocks 3 and 4 are not too promising. It may have been caused by the small number of subjects in this study; thus, they could not reliably detect an improvement in performance. A large variation in the results after AT in these swimmers also contributed to this problem. This phenomenon was repeated in all the cited observations on swimmers.

## Conclusions

Literature data analysis shows that cardiovascular-respiratory efficiency index of maximal minute oxygen uptake (V̇O_2max_) decreases by 0.9% for every 100m above an altitude of 1100 m above sea level (Roberts et al. 1998, Wehrlin et al. 2006, Vogt et al. 2010). That is why during training in natural mountain conditions this relative lowering of endurance should be taken into consideration, which may be directly transferred to the necessity of swimming speed reduction in order to equalize intensity in a given physical effort in comparison to exercise at sea level. Limited oxygen availability during recovery should also be a factor that inclines AT intensity reduction, as for example in the above mentioned AT by Truijens et al. (2003).

In this review AT in its variety was mainly conducted in 3-week periods, from less than 2 weeks (Robach et al., 2006) *LH+TL* with hypoxia stimulation increased after 5 days from 2500 m to 3000 m above sea level, to almost 6 weeks in four 10-day blocks using *LH+TL* and traditional (*LH+TH*) (Robertson et al., 2010). Such periodical repetition of stimulation using *LH+TH* should stimulate haematological adaptive changes. Furthermore, hypoxia dosage in the IHE and IHT, technical reasons and apnoea training facilitate the anaerobic endurance component and maintains aerobic endurance adaptation while, together with normoxic training, enables the development of race pace efficiency.

Swimmers who train with hypoxia should be among the best-trained athletes, in which even a slight improvement in physical endurance might mean improvement of the swimming time in a given competition. It should be taken into consideration that such stimulation of very well-trained swimmers, close to their *plateau* level, might make a negligible contribution when compared to a non elite group. But even an improvement in results which is statistically insignificant, for example by 2 seconds in 400 m swimming, may mean a record breaking or medal winning time in national, European or world championships. In the discussed research on swimmers using the AT method, the achievement of individual results that were beyond expectation could also be seen. It may apply to athletes which have an appropriate reserve susceptible to training. If so, coaches should concentrate on activities that aim to discover such predispositions in athletes. It is presumed that hard to define reserves slip out of known prediction methods because it implies to a particular characteristic for each subject’s gene expression. On the other hand, for children and adolescents who have potential, which could be stimulated using conventional methods, AT should not be included.

The review of the available research indicates that even in elite athletes, high mountain conditions or its artificially evoked equivalent, do not always bring the desired results. That is the reason why AT training still needs further research on homogenous groups of athletes with a deliberate attempt to explain highly variable indices. Nonetheless, properly conducted AT and its variations may be and are one of the methods of achieving the highest level of endurance and improvement in performance.

## Figures and Tables

**Table 1a.** A summary of studies on altitude and hypoxic training with swimmers.

Authors	Hypoxic Group	Type of training	Research schedule	Post-altitude and altitude of swimming, hematological, biochemical, endurance results (↑) improment, (→) no change, (↓) deterioration
	Control Group			
Miyashita et al. (1988)	8♂, (13–19y)	**LH+TH**	21 day swimming training at altitude 2300m (Mexico City, Mexico)	→V competition 100m, ↑ V competition 200m, ↑ RBC, →Hb, ↑Htc
	no			
	4♀, 8♂ (13–18y)			↑RBC, ↑Hb, ↑Htc, V (not measured)
	no			
Martino et al. (1995)	12♀, 8♂		21 day sprint swimming training at altitude 2800m	↑V 100m; ↑peak power [W] and →mean power [W] of upper extremities in Wingate test. No significant improvement in the control group
	7♀, 6♂			
Pyne (1998)	8♀, 14♂		24 day swimming training camp at altitude 2100m (Flagstaff, USA)	↑V max in 5x200m test, →La/V (lactate-velocity curve in 5×200m test)
	no			
Tachi et al. (2002)	3♀, 3♂, (18.0±1.3y)		Two separate altitude swimming camps with 1,5 month gap, at 2100m (Flagstaff, USA)First held 21 day, second 19, 21 or 30 day for each pair of swimmers	↑(ns) shift to the right La/V of lactate-velocity curve in incremental swimming tests through and after both camps
	no			
Roels et al.. (2006)	8♂ (16.3±0.9y)		Two 13 days camps separated by 6 weeks of sea level training. First camp held on ≈1200m, second camp held on ≈1850m	Camp I: 1200m: ↑sTfR, →MCV, ↓Reticulocytes [%], →RBC, →V_Emax_, →VO_2max_, ↑V 2000m. Camp II: 1850m: ↑ sTfR, ↑ MCV, ↑Reticulocytes [%], → RBC, →V_Emax_, →VO_2max_, →V 2000m
	no			
Ogita et al. (1999)	9♂, (20.0±1.0y)	**IHT**	High intensity intermittent training under hypoxic hypobaric condition on ≈3000m, 2h d^−1^·5d · wk ^−1^·2 wk	↑anaerobic capacity-MAOD, ↑time and velocity of submaximal swimming in flume, →VO_2max_
	no			
Ogita (2006)	6♂		The training under hypoxic hypobaric conditions for 2 sessions daily · 5d · wk ^−1^·3 wk on ≈1600m and ≈2400m	↑V100, ↑V200, ↑VO_2max_, ↑anaerobic capacity –MAOD (improvement of both indices)
	6♂			
Truijens et al. (2003)	5♀, 3♂, (28.8±12.1y)		High-intensity interval training sessions under hypoxia in a flume 3d·wk^−1^· 5 wk on ≈2500m and supplemental low or moderate-intensity sessions under normoxia in a pool	↑V 100m and ↑V 400m freestyle as well as ↑VO_2max_ and ↑V_Emax_ in both hypoxic and placebo groups, → anaerobic capacity –MAOD, → swimming economy, →Hb, →Htc
	5♀, 3♂, (28.9±9.2y)			

♀ *– woman;* ♂ *– man; LH+TH – live high and train high; V – swimming speed [m·s*^−^*^1^]; RBC – red blood cell count [10*^−^*^6^ ·μL*^−^*^1^]; Hb – haemoglobin [g·dL*^−^*^1^]; Htc – hematocrit [%]; sTfR – soluble transferrin receptor [nmol·l*^−^*^1^]; MCV – mean corpuscular volume [fl]; V_Emax_ – maximal ventilation [l·min*^−^*^1^]; VO_2max_ – maximal oxygen uptake [l·min*^−^*^1^]; ns – statistically nonsignificant; IHT – intermittent hypoxic training; MAOD – maximal accumulated O_2_ deficit.*

**Table 1b.** A summary of studies on altitude and hypoxic training with swimmers.

Authors	Hypoxic Group		Type of training	Research schedule	Post-altitude and altitude of swimming, hematological, biochemical, endurance results (↑) improment, (→) no change, (↓) deterioration
	Control Group				
Rodríguez et al. (2003)	8♀, ♂		**IHE**	2-weeks sea level training combined with 3h·d^−1^ at simulated altitude ≈4000–5500m	→100m, ↑V 200m, ↑VO_2peak_, ↑VO_2max_ in swimming 200m test, ↑Hb, ↑RBC, ↑Retikulocytes [%], ↑Htc
	8♀, ♂				
Rodríguez et al. (2007)	6♀, ♂	(20.2±8.8y)		Through 4 weeks 3h d^−1^·5d· wk^−1^ in a hypobaric chamber (IHE). It simulated altitude: in 1st–2nd day on ≈4000m, 3th–4th day on ≈4500m, 5th–6th day on ≈5000m and from 7th up to 20th day on ≈5500m	1 week after IHE: →T-100m, → T-400m in both groups → VO_2max_, →V_Emax_, ↑VO_2_-VT. 3 weeks after IHE: →T-100m, → T-400m, ↑ VO_2max_ (p<0.07), ↑V_Emax_, ↑ VO_2_-VT
	7♀, ♂				
Robach et al. (2006)	1♀, 9♂ (17.0±0.5y)		**LH+TL (IHE)**	Hypoxic (H) and control (C) group of swimmers trained at an altitude 1200m, H group was exposed 16h·d^−1^·13d in hypoxic room. For 5 days they breathed hypoxic air ≈2500m, than next 8 days ≈3000m	H group →T-2000m, →Vmax in 4×200m test, ↑V_Emax_, ↑Hb, ↑VO_2max_ (p=0.09). During IHE: ↑EPO, ↑Reticulocytes [%], ↑sTfR, →Ferritin [μg·l^−1^], →Htc. After 15 days any significant changes was noted. C group: ↑V_max_ in 4×200m test after two weeks, ↑T-2000m after 15 days.
	1♀, 9♂ (20.0±3.0y)				
Lemaître et al. (2009)	4♀,♂ (20.5±1.7y)		**Apnoea**	For 3 consecutive months, 1-hour apnoea training sessions were held 3 times a week.	→V 50m, (→ La_peak_, ↑VO_2peak_, ↑SaO_2_min, ↑RCP in maximal incremental test on cycle ergometer)
	no				
Friedmann et al. (2005)	9♀, 7♂ (16.4±1.4y)		**Combined**	Initially swimmers were exposed 4h to normobaric hypoxia-IHE ≈2500m, then they participated for 3 weeks in altitude camp LH+TH at alt ≈2100m to ≈2300m Sierra Nevada (Spain).	↑EPO after 4h IHE as well after first and second day of LH+TH, ↑ 2–3% swimming with OBLA velocity and Vmax in 5×100m and 5×400m tests, ↑THM, → Hb, →Htc
	no				
Robertson et al. (2010)	4♀, 5♂	(21.1±3.0y)		Three to four 10-day blocks: 1 i 3 block LH+TL (≈2600m nights –IHE 9-10h + 600m with training). 2 and 4 day blocks LH+TL consisted 5 nights – IHE 9–10h ≈2600m + 600m with training, followed by 5 days of living and training in Thredbo 1350m, Australia.	↑THM (ns), ↑VLT, ↑T-2000m freestyle and T-1200m breaststroke after two blocks. →swimming results after block 3 in the National Championships and after block 4 in the Commonwealth Games). No substantial difference in mean improvement between the groups in both competitions
	3♀, 6♂				

♀ *– woman;* ♂ *– man; IHE – intermittent hypoxic exposure at rest and/or sleep; V – swimming speed [m·s*^−^*^1^]; VO_2peak_ - peak oxygen uptake [l·min*^−^*^1^]; Hb – haemoglobin [g·dL*^−^*^1^]; Htc – hematocrit [%]; T-100m – time trial performance at the distance of 100m [s]; T-400m – time trial performance at the distance of 400m; VO_2max_ – maximal oxygen uptake [l·min*^−^*^1^]; V_Emax_ – maximal ventilation [l·min*^−^*^1^]; VO_2-VT_– oxygen uptake at ventilatory threshold ; LH+TL – living high and train low; T-2000m time in endurance test at the distance of 2000 freestyle; EPO – erythropoietin [mU·mL*^−^*^1^]; sTfR – soluble transferrin receptor [nmol·l*^−^*^1^]; La_peak_ – peak blood lactate accumulation [mM]; SaO_2_min – minimal arterial oxygen saturation [%]; RCP– power on respiratory compensation point [W]; LH+TH – live high and train high; OBLA – onset of blood lactate accumulation; THM – Total haemoglobin mass measured with CO-rebreathing method; V_LT_ – threshold of blood lactate accumulation; ns – statistically nonsignificant; T-2000m and T-1200m – time in endurance tests at the distance of 2000 m and 1200m [s].*
